# Living with severe allergy: an Anaphylaxis Campaign national survey of young people

**DOI:** 10.1186/2045-7022-3-2

**Published:** 2013-01-22

**Authors:** Allison Worth, Lynne Regent, Mark Levy, Carey Ledford, Mandy East, Aziz Sheikh

**Affiliations:** 1 Allergy & Respiratory Research Group, Centre for Population Health Sciences, The University of Edinburgh, Medical School, Teviot Place, Edinburgh EH8 9AG, UK; 2 The Anaphylaxis Campaign, PO Box 275, Farnborough, Hampshire GU14 6SX, UK

**Keywords:** Allergy, Anaphylaxis, Young people

## Abstract

**Background:**

The transition to adulthood can be particularly challenging for young people with severe allergies, who must learn to balance personal safety with independent living. Information and support for young people and their families are crucial to successfully managing this transition. We sought to: gather insights into the impact of severe allergies on the lives of young people; explore where young people go for information about anaphylaxis and what information they want and need; identify areas where further support is needed.

**Methods:**

An online questionnaire survey of young people aged 15–25 years with severe allergies in the United Kingdom (UK) was conducted on behalf of the Anaphylaxis Campaign, the main patient support organisation. Participants were recruited mainly from the Anaphylaxis Campaign membership database and also via allergy clinics and social media. The study was funded by the Anaphylaxis Campaign’s In Memoriam Fund.

**Results:**

A total of 520 young people responded to the survey. The majority had lived with severe allergies since they were young children; 59% reported having attended Accident and Emergency units as a consequence of their allergies. Only 66% of respondents reported always carrying their epinephrine auto-injectors; only 23% had ever used these. Few were currently receiving specialist allergy care; younger respondents were more likely to be under specialist care (34%) than those 18 years and above (23%). Respondents wanted more information about eating out (56%), travelling (54%) and food labelling (43%). Almost a quarter of respondents (23%) reported needing more information on managing their allergies independently without parental help. Managing allergies in the context of social relationships was a concern for 22% of respondents.

**Conclusions:**

This survey has identified the information and support needs and gaps in service provision for young people with severe allergies. Healthcare professionals and patient support organisations, with the support of the food industry, can help to meet these needs.

## Background

Adolescents with severe allergies are recognised as a high-risk group for severe and fatal reactions [1-4]. The pronounced psychosocial impact on individuals with severe allergies and their families is also well-established [5-8]. Foods are the dominant triggers for anaphylaxis in young people [9]. Recent studies have highlighted the difficulties adolescents have in managing their allergies and inconsistencies in provision of services, which should help them to manage their allergies more effectively [10-15]. Adolescents appear to find risks difficult to judge, particularly with regard to eating away from home, and have difficulty accessing and interpreting information on bought and catered food, leading some adolescents to take risks, for example by eating foods which ‘may contain nuts’ [10,14,15], or perceiving that carrying their epinephrine auto-injectors allows them to take risks with potentially unsafe food [1,16]. Young people are also known to lack confidence in recognising and managing severe allergic reactions and under-use epinephrine (adrenaline) auto-injectors [17,18]. These factors have led to calls for improved educational interventions to support more effective self-management [4,16,17,19].

Adolescence is characterised by growing independence, expanding social horizons and a move to autonomous decision-making [20]; young people with severe allergies and their parents would therefore benefit from support and advice in managing this transition [12,14]. What is lacking in the literature to date are data providing insights into whether the problems and challenges identified through in-depth qualitative work [10-14], with relatively small numbers of patients, are of concern to the wider pool of people living with severe allergies.

As the main UK patient support charity, the Anaphylaxis Campaign aims to provide information, support and education to people at risk of anaphylaxis, their families, health professionals and industry. The Campaign has identified young people at risk of anaphylaxis as a group with particular support needs and the survey was instituted to gather information to support development of a specific youth programme.

Our main objective was to gather insights into the impact that severe allergies have on the lives of young people. Secondary objectives were to explore where young people seek information about anaphylaxis and what information they want or need, in order to inform future activities by the Anaphylaxis Campaign, and to identify areas where further support is needed.

## Methods

### Design

An online questionnaire survey was undertaken in order to reach a large number of young people with severe allergies from across the UK.

### Recruitment

The target group, young people with severe allergies aged 15–25 years who had been prescribed an epinephrine auto-injector, were contacted primarily through a database of parents who are signed up to receive the Anaphylaxis Campaign newsletter. All parents of young people aged 15 years, and all 16–25 year olds were contacted via email (n = 1317) or, if no email address was available, by post (n = 1175). Information about the research was also posted on the Anaphylaxis Campaign’s website with a link to the survey.

In order to reach potential participants who/whose parents might not have been members of the Anaphylaxis Campaign, information on the survey was sent to 48 allergy clinics throughout the UK and Channel Islands, with a request to publicise the survey to eligible patients. The Campaign also placed posts on its Facebook and Twitter pages, asking potential participants to complete the survey and/or pass the information on to anyone they knew who might qualify for the survey. Parental consent was sought for respondents aged 15, as is required for those under the age of 16 to participate in market research.

### Data generation

The survey was conducted on behalf of the Anaphylaxis Campaign by ComRes, a market research agency, between February and April 2012. The Anaphylaxis Campaign drafted the questions based on their knowledge of circumstances which young people may find challenging or concerning regarding their allergies. ComRes utilised their market research expertise to suggest suitable wording of questions and The Anaphylaxis Campaign ensured the correct language and medical terminology were used. The questionnaire was piloted with six young people and minor changes were made based on their feedback.

The final questionnaire is provided in Additional file 1. Participants completed the survey online; this required an estimated 5–10 minutes to complete.

### Data analysis

Descriptive analysis was conducted by ComRes in consultation with The Anaphylaxis Campaign. Further analysis, including interpretation of findings and comparisons with existing literature, was undertaken by AW and AS in consultation with The Anaphylaxis Campaign.

### Ethics

The study was conducted in keeping with the Market Research Society Code of Conduct and British Polling Council guidelines which commit ComRes to ethical and transparent practices.

## Results

A total of 520 young people with severe allergies completed the survey.

### Characteristics of respondents

**Figure 1:**
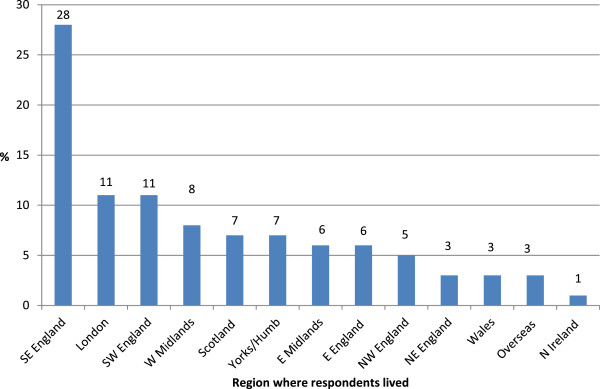
Region where respondents lived (%).

**Figure 2:**
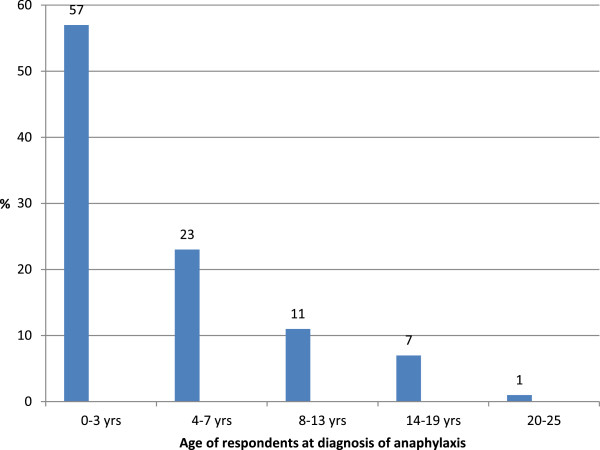
Age of respondents at diagnosis of anaphylaxis (%).

The mean age of respondents was 18.5 years (SD 2.8); 43% of respondents were aged 15–17 years (n = 223), 40% aged 18–21 years (n = 209) and 17% aged 22–25 years (n = 88). Responses were received from young people from each of the four nations of the UK (Figure 1). The majority reported being diagnosed with anaphylaxis before the age of eight (Figure 2) and their responses indicate that food was their main trigger. The vast majority of respondents lived with their parents (79%), while almost one in five lived at university with others (18%). A further 12% lived either with friends or with their partner and 4% lived alone (respondents could tick all that apply, so some ticked both ‘living with parents’ and ‘living at university’).

### Accident & emergency attendances

The majority of respondents (59%) reported that they had been taken to an Accident & Emergency unit (A&E) as a result of their allergies, while a third (34%) said that they had not and 7% were unable to remember.

### Epinephrine auto-injector carriage and use

All respondents reported being prescribed an epinephrine auto-injector at some point. Only 66% reported carrying their auto-injector everywhere they go, with a further 28% reporting they carried it most places. Six per cent of respondents said that they rarely or never carried their auto-injector.

Three-quarters (77%) of respondents said that they had never had to use their auto-injector – this was highest among younger respondents (82% of those aged 15–17, compared to 74% of those aged 18–25). Around one in 10 (11%) had used their auto-injector once, and 12% reported that they had used their auto-injectors twice or more.

Half (51%) of respondents who reported never having used their epinephrine auto-injector also reported that they had been to A&E as a result of their allergies. Despite the use of epinephrine auto-injectors being limited among young people, their reported confidence in their ability to self-administer epinephrine was relatively high. The majority (77%) of respondents said they were confident about using their auto-injector, while around a quarter (23%) said that they were not confident. Older respondents were more likely to be confident about self-administering epinephrine than younger respondents – 80% of respondents aged 18–25 said they were confident, compared to 74% of those aged 15–18.

Respondents who lived away from their parents expressed slightly more confidence than respondents who lived with their parents about self-administering epinephrine: 75% of those who lived with their parents said that they were either ‘very’ or ‘fairly’ confident compared with 83% of those who lived away from their parents with others.

### Specialist care

Only 28% of respondents were currently under the care of an allergy specialist, while nearly half (47%) reported that they were not currently under the care of an allergy specialist, but had been in the past. A quarter (24%) of respondents reported that they had never been under the care of an allergy specialist.

### Allergy management

Respondents were asked to write in three things that they do to manage their allergies. The eight most common responses are listed in Table 1.

**Table 1:** Strategies for allergy management

**Allergy management strategy**	**N (%)**
Check food labelling/ingredients/packaging	289 (56)
Carry an epinephrine auto-injector	208 (40)
Avoid food containing nuts/avoid nuts	127 (24)
Educate family/friends/make sure everyone is aware	127 (24)
Take care when eating out/in restaurants	115 (22)
Monitor/check everything I eat/careful about what I eat	112 (22)
Carry medication/antihistamine	81 (16)
Avoid food containing allergens/what I’m allergic to	49 (9)

### Main concerns about allergy

Participants were asked to name the one thing that concerned or bothered them most about their allergy (Table 2). Overall, 37% of respondents said that the one thing that concerned or bothered them most was to do with their ability to eat, their diet, or going to restaurants. Concerns regarding relationships or social life were mentioned by 22% of respondents. More than one in 10 respondents (12%) reported their main concern was the prospect of an anaphylactic reaction, and 8% were most concerned about the risk of fatality.

**Table 2:** Main concerns regarding allergy

	**N (%)**
Restricted diet/can’t try different food/restaurants	61 (12%)
Worried about having an anaphylactic shock/reaction (when out) and not being able to deal with it/nobody to help	61 (12%)
Having to constantly ask/check/find out what foods contain/whether contain nuts etc.	51 (10%)
Life threatening condition/could be fatal/serious	40 (8%)
Can’t eat same as friends/family/have to order different things	36 (7%)
Other people’s ignorance/lack of understanding	34 (7%)
Concerns with food packaging/labelling (too vague, too cautious etc.)	29 (6%)
Restricts/limits my life	24 (5%)

### Main effects of having an allergy

The most commonly cited effects of having a severe allergy are presented in Table 3.

**Table 3:** Main effects of having an allergy

	**N (%)**
Carry an epinephrine auto- injector	444 (85)
Difficulty travelling/going on overnight trips	239 (46)
I have found it difficult socialising and going out with friends/found it difficult making friends/getting a boyfriend or girlfriend	202 (39)
My parents have become over-protective of me	143 (28)
Restricted diet/problems with eating out	97 (19)
I found it difficult moving away from home	47 (9)

### Moving away from home

The 160 respondents who had left home were asked to select the three hardest things about moving away. The three most selected options were: the need to check the food they eat and buy (19%); explaining to new housemates and friends (15%); and not having parents around to provide support and help (14%).

### Sources of information about anaphylaxis

Respondents were asked where they would seek information about anaphylaxis and reported a range of potential sources. Most said they would seek information from a healthcare professional of some kind, either their GP (53%), or allergy clinic (38%); interestingly, this is more than were actually under the care of an allergy clinic. Among those still at school, 14% of respondents aged 15 to 17 would go to the school nurse. The Anaphylaxis Campaign was viewed as a source of information by 72% of respondents and friends or family by 39%.

### Information needs

The main areas where respondents reported needing more information were eating out (56%), travelling (54%) and food labelling (43%). Older respondents were more likely to require information about travelling: 60% of 18–25 year olds, compared to 46% of 15–17 year olds.

Almost a quarter of respondents (23%) reported needing more information on managing their allergies independently without the help of their parents. As might be expected, younger respondents were more likely to say that they need this sort of information – a third of all respondents aged 15–17 (35%) said this, compared to 13% of those aged 18–25.

Respondents were least likely to say that they needed additional information on their epinephrine auto-injector – just 18%. However, this is associated with the level of confidence that respondents reported having regarding the use of their injector: 41% of those who were not confident said that they required more information, compared to just 12% who were confident self-injecting using their epinephrine auto-injector.

Respondents were asked to rank six sources of information in terms of how useful they would find them (Table 4). General and medical information were seen as the most useful. There was a range of views on the value of web-based services, which were not seen as one of the most useful options, although few respondents regarded it as the least useful. Similarly, downloadable resources such as mobile phone applications were seen as useful by some respondents, but not all. For example, older respondents (those aged 22–25) were most likely to find these useful (20%). However, 21% of all respondents said that this was the ‘least’ useful option.

**Table 4:** Level of usefulness of different forms of information

**Form of information**	**Mean scores (SD)**
General tips and advice to deal with severe allergies	2.74 (1.49)
Medical information surrounding anaphylaxis	3.09 (1.53)
Web-based services for young people living with anaphylaxis	3.17 (1.38)
An online forum (where young people can talk to others with similar severe allergies)	3.55 (1.77)
Downloadable resources such as mobile phone applications	3.71 (1.79)
Someone to talk to (i.e. meet up face to face) about your concerns	4.66 (1.58)

1 = most useful and 6 = least useful.

## Discussion

### Summary of main findings

This study provides valuable insights into how young people manage severe allergy and the impact it has on their lives. A high proportion of children/young people with severe allergies have received emergency care, relatively few are under the care of a specialist, epinephrine carriage and use is sub-optimal, and there is a perceived need for support in managing everyday life issues such as eating out, travel and social management. The majority of respondents lived at home, reflecting current social trends, and emphasising the continuing importance of the family in anaphylaxis prevention and management even as adulthood is achieved.

The high proportion of respondents who reported having had to attend A&E as a consequence of their allergy suggests an at-risk group for severe allergic reactions, yet only 28% reported receiving specialist care and this proportion decreased after 17 years, suggesting transition to adult services remains inadequate. While many respondents reported acting responsibly with regard to carrying epinephrine auto-injectors, only 66% carried them all the time, contrary to usual healthcare professional/Anaphylaxis Campaign advice. Few had used their auto-injector; given the number of A&E visits, this may indicate that young people may not have used their auto-injector when they should. Although the majority of respondents expressed confidence in self-administering epinephrine, almost one quarter did not, particularly in the youngest respondents, indicating a training need; confident use of emergency medication is one of the cornerstones of good anaphylaxis management. This is particularly concerning as the respondents’ parents were mostly Anaphylaxis Campaign members, who could be expected to be well-informed and have access to better information than non-members.

Issues surrounding diet and eating practices were the biggest challenge identified by respondents. Social aspects, including forming relationships, explaining allergies to new friends and leaving home were also challenging, indicating that allergies add an additional problem to the normal teenage transitions associated with becoming independent.

Information needs were identified, with both general and medical advice on allergy management required. Interestingly, young people were divided on the value of web-based and downloadable information (e.g. mobile phone applications), with the strongest support coming from those aged 22–25 years, but even then this was not particularly convincing.

### Strengths and limitations

The large number of young people who responded reflects the importance attached to the topic by young people with severe allergies. The study usefully provides corroborative data which largely support and complement findings from previously conducted qualitative studies [10-14,21], confirming the widespread nature of the problems identified.

The chief limitation of the study is the difficulty in fully describing the study population: data on sex, ethnicity and type of allergy were not collected and there was no objective means of confirming a diagnosis of severe allergy beyond the prescription of an epinephrine auto-injector. It is not possible to say what proportion of respondents came from the various sources of recruitment, nor what proportion were members or past members of the Anaphylaxis Campaign. It is likely that the majority of respondents were children of Anaphylaxis Campaign members and the responses suggest they were predominantly food allergic; the generalisability of findings is therefore difficult to assess as respondents may have been more knowledgeable about allergy and received more support than non-members’ children. However, in the absence of any national sampling frame it was necessary to use a convenience-based sampling strategy; the survey provided broad age, gender and geographical coverage, but care should still be taken in extrapolating beyond the study population. The findings also rely on self-report and recall; young people may report their behaviour more positively than realistically, with some impact on the reliability of the data.

### Implications for future research and clinical practice

This work indicates a number of areas where young people with severe allergies need more support and information. The fact that 25% of these respondents had never received specialist care is alarming, given the potential for life threatening episodes of anaphylaxis. This might be provided by allergy services, primary care, school nurses and patient support organisations such as the Anaphylaxis Campaign. Support might usefully focus on preparing young people and parents for the time when the young person leaves home, so that young people are better prepared for the practical and social challenges of managing allergies. Information and practical advice, tailored to individual circumstances, could support more effective self-management [4,17,21].

## Conclusions

This survey has identified the information and support needs and gaps in service provision for young people with severe allergies. In particular, young people need support in preparation for the transition to independent living. Healthcare professionals and patient support organisations can help to meet these needs and also to expedite specialist supervision where appropriate and available. Although online information and mobile phone applications may provide back-up, the respondents in our survey suggested that face-to-face discussions are the most helpful way to receive information and support.

## Abbreviation

A&E: Accident & Emergency unit.

## Competing interests

AW has received support from ALK-Abello and Napp to attend conferences in the previous 5 years. AS has received support from ALK-Abello, Lincoln Medical, Meda, Napp and Thermo Fisher to attend educational meetings, conferences and advisory work in the previous 5 years.

## Authors’ contributions

LR, CL and ME conceived and designed the study and contributed to data analysis; AW, AS and ML contributed additional interpretation of the data; AW drafted the manuscript, with contributions from all authors; all authors read and approved the final manuscript.

## Authors’ information

LR is Chief Executive of The Anaphylaxis Campaign, CL is Membership and Community Fundraising Manager of The Anaphylaxis Campaign and ME is National Coordinator of The Anaphylaxis Campaign. ML is Chairman of the Scientific Advisory Group of the Anaphylaxis Campaign. AS leads and AW manages the Allergy and Respiratory Research Group at The University of Edinburgh. AS was a member of the Royal College of Paediatrics and Child Health’s Care Pathway for Children at Risk of Anaphylaxis, is a member of the UK Resuscitation Council Anaphylaxis Guidelines Committee, the World Allergy Organization’s Special Committee on Anaphylaxis, the European Academy of Allergy and Clinical Immunology’s Steering Committee of the Food Allergy and Anaphylaxis Guidelines, the Scientific Committee of the Anaphylaxis Campaign and is the (joint) Royal College of General Practitioners Clinical Champion for Allergy.

## Supplementary Material

Additional file 1 Survey questionnaire. Click here for file

## References

[B1] Sampson MA Muñoz-Furlong A Sicherer SH Risk-taking and coping strategies of adolescents and young adults with food allergy J Allergy Clin Immunol 2006 117 1440 1445 http://dx.doi.org/10.1016/j.jaci.2006.03.009 10.1016/j.jaci.2006.03.009 16751011

[B2] Bock SA Muñoz-Furlong A Sampson HA Further fatalities caused by anaphylactic reactions to food, 2001–2006 J Allergy Clin Immunol 2007 119 1016 1018 http://dx.doi.org/10.1016/j.jaci.2006.12.622 10.1016/j.jaci.2006.12.622 17306354

[B3] Pumphrey R Gowland H Further fatal allergic reactions to food in the United Kingdom, 1999–2006 J Allergy Clin Immunol 2007 119 1018 1019 http://dx.doi.org/10.1016/j.jaci.2007.01.02 10.1016/j.jaci.2007.01.021 17349682

[B4] Marrs T Lack G Why do few food-allergic adolescents treat anaphylaxis with adrenaline? – reviewing a pressing issue Pediatr Allergy Immunol 2012 Epub ahead of print] http://dx.doi.org/10.1111/pai.12013 10.1111/pai.12013 23173610

[B5] Mandell D Curtis R Gold M Hardie S Families coping with a diagnosis of anaphylaxis in a child ACI International 2002 14 96 101 http://dx.doi.org/10.1027/0838-1925.14.3.96

[B6] Lyons AC Forde EME Food allergy in young adults: perceptions and psychological effects J Health Psychol 2004 9 497 504 http://dx.doi.org/10.1177/1359105304044032 10.1177/1359105304044032 15231052

[B7] Cummings AJ Knibb RC King RM Lucas JS The psychosocial impact of food allergy and food hypersensitivity in children, adolescents and their families: a review Allergy 2010 65 933 45 http://dx.doi.org/10.1111/j.1398-9995.2010.02342.x 10.1111/j.1398-9995.2010.02342.x 20180792

[B8] Cummings AJ Knibb RC Erlewyn-Lajeunesse M King RM Roberts G Lucas JS Management of nut allergy influences quality of life and anxiety in children and their mothers Pediatr Allergy Immunol 2010 21 586 94 http://dx.doi.org/10.1111/j.1399-3038.2009.00975.x 10.1111/j.1399-3038.2009.00975.x 20088863

[B9] Alves B Sheikh A Age-specific aetiology of anaphylaxis: a study of routine hospital admission data in England Arch Dis Child 2001 85 349 http://dx.doi.org/10.1136/adc.85.4.348b 10.1136/adc.85.4.349 PMC1718933 11572233

[B10] Akeson N Worth A Sheikh A The psychosocial impact of anaphylaxis on young people and their parents Clin Exp Allergy 2007 37 1213 1220 http://dx.doi.org/10.1111/j.1365-2222.2007.02758.x 10.1111/j.1365-2222.2007.02758.x 17651152

[B11] Marklund B Wilde-Larsson B Ahlstedt S Nordström G Adolescents’ experiences of being food-hypersensitive: a qualitative study BMC Nursing 2007 6 8 http://dx.doi.org/10.1186/1472-6955-6-8 10.1186/1472-6955-6-8 17922926 PMC2104527

[B12] Mackenzie H Roberts G van Laar D Dean T Teenagers’ experiences of living with food hypersensitivity: a qualitative study Pediatr Allergy Immunol 2010 21 595 602 http://dx.doi.org/10.1111/j.1399-3038.2009.00938.x 19702674 10.1111/j.1399-3038.2009.00938.x

[B13] Monks H Gowland MH MacKenzie H Erlewyn-Lajeunesse M King RM Lucas JS Roberts G How do teenagers manage their food allergies? Clin Exp Allergy 2010 40 1533 40 http://dx.doi.org/10.1111/j.1365-2222.2010.03586.x 10.1111/j.1365-2222.2010.03586.x 20682004

[B14] Gallagher M Worth A Cunningham-Burley S Sheikh A Strategies for living with the risk of anaphylaxis in adolescence: qualitative study of young people and their parents Prim Care Resp J 2012 21 392 7 http://dx.doi.org/10.4104/pcrj.2012.00072 10.4104/pcrj.2012.00072 PMC6548038 22875142

[B15] Greenhawt MJ Singer AM Baptist AP Food allergy and food allergy attitudes among college students J Allergy Clin Immunol 2009 124 323 7 http://dx.doi.org/10.1016/j.jaci.2009.05.028 10.1016/j.jaci.2009.05.028 19560802

[B16] Lockey RF Lockey RF Adolescents and anaphylaxis Prim Care Resp J 2012 21 365 6 http://dx.doi.org/10.4104/pcrj.2012.00090 10.4104/pcrj.2012.00090 PMC6548055 23090435

[B17] Gallagher M Worth A Cunningham-Burley SC Sheikh A Epinephrine auto-injector use in adolescents at risk of anaphylaxis: a qualitative study in Scotland, UK Clin Exp Allergy 2011 41 869 77 http://dx.doi.org/10.1111/j.1365-2222.2011.03743.x 10.1111/j.1365-2222.2011.03743.x 21481022

[B18] Noimark L Wales J Du Toit G Pastacaldi C Haddad D Gardner J Hyer W Vance G Townshend C Alfaham M Arkwright PD Rao R Kapoor S Summerfield A Warner JO Roberts G The use of adrenaline auto-injectors by children and teenagers Clin Exp Allergy 2012 42 284 92 http://dx.doi.org/10.1111%2Fj.1365-2222.2011.03912.x 10.1111/j.1365-2222.2011.03912.x 22181034

[B19] Money AG Barnett J Kuljis J Lucas J Patient perceptions of epinephrine auto-injectors: exploring barriers to use Scand J Caring Sci 2012 [Epub ahead of print] http://dx.doi.org/10.1111/j.1471-6712.2012.01045.x 10.1111/j.1471-6712.2012.01045.x 22834703

[B20] Christensen P Mikkelsen MR Jumping off and being careful: children’s strategies of risk management in everyday life Sociol Health Illness 2008 30 112 130 http://dx.doi.org/10.1111/j.1467-9566.2007.01046.x 10.1111/j.1467-9566.2007.01046.x 18254836

[B21] Macadam C Barnett J Roberts G Stiefel G King R Erlewyn-Lajeunesse M Holloway JA Lucas JS What factors affect the carriage of epinephrine auto-injectors by teenagers? Clin Transl Allergy 2012 2 3 10.1186/2045-7022-2-3 22409884 PMC3299626

