# Supplementary material for: Living with severe allergy: an Anaphylaxis Campaign national survey of young people
Source: Clin Transl Allergy. 2013 Jan 22;3:2. doi: 10.1186/2045-7022-3-2 (PMC3560150; doi:10.1186/2045-7022-3-2)
Supplement: Additional file 1 — Survey questionnaire. [file 2045-7022-3-2-S1.doc]

**Appendix 1: Survey questionnaire**

Welcome to the Anaphylaxis Campaign’s Young People’s Survey.  You have been invited to take part in this survey because we are interested to hear about your experiences as a young person living with anaphylaxis.

- Please be reassured that all of your responses will remain confidential.
- This survey should take no longer than 10 minutes to complete.
- We will enter all participants into a prize draw to win £100.
- Feel free to contact Nick Barratt at ComRes on 0207 871 8666 or on [nick.barratt@comres.co.uk](mailto:nick.barratt@comres.co.uk.).
- ComRes conducts all our surveys in accordance with the Market Research Society's Code of Conduct and therefore all your responses will be strictly anonymous.

**Anaphylaxis survey**

Q1. How old are you?

- 15
- 16
- 17
- 18
- 19
- 20
- 21
- 22
- 23
- 24
- 25
- None of the above

Q2. Which region do you live in?

- Scotland
- North East
- North West
- Yorkshire & Humberside
- West Midlands
- East Midlands
- Wales
- East of England
- London
- South East
- South West
- Northern Ireland
- Overseas

Q3. How old were you when you were first diagnosed with having anaphylaxis?

- 0-3
- 4-7
- 8-10
- 11-13
- 14-16
- 17-19
- 20-22
- 23-25
- Don’t know

Q4. Have you been prescribed an adrenaline injector, also known as an EpiPen, AnaPen or Jext at any time of your life?

- Yes
- No

Q5. Which of the following applies to you? Please tick all that apply.

- I live with my parents
- I live by myself
- I live at university alone
- I live at university with others
- I live with friends
- I live with my partner

Q6. How often would you say that you have had to use your adrenaline injector?
(If you are unsure please make an educated guess)

- I have never had to use it
- Once
- Twice
- 3-5 times
- 5-10 times
- More than 10 times
- Don’t know/ Can’t remember

Q7. Have you ever been taken to A&E as a result of your allergies?

- Yes
- No
- Don’t know/ Can’t remember
- I would prefer not to say

Q8. Other than your GP, are you under the care of an allergy specialist (at a hospital clinic)?

- Yes, I am currently under the care of an allergy specialist
- No, I am not currently, but I have been under the care of an allergy specialist in the past
- I have never been under the care of an allergy specialist
- I would prefer not to say

Q9. Please write in up to three things that you do to manage your allergies.

| 1 | ______________________________ |
| --- | --- |
| 2 | ______________________________ |
| 3 | ______________________________ |

Q10. How confident or otherwise do you feel about giving yourself an adrenaline injection, assuming that you were well enough to do so?

- Very confident
- Fairly confident
- Not very confident
- Not at all confident

Q11. Which ONE of the following statements best applies to you?

- I carry my adrenaline injector everywhere I go
- I carry my adrenaline injector most places that I go, but not everywhere
- I don’t often carry my adrenaline injector with me
- I never carry my adrenaline injector with me

Why don’t you carry your adrenaline injector with you? Please write your reasons in the box below

|  |
| --- |

Q12. Please write in the ONE thing that most concerns or bothers you about your allergy.

|  |
| --- |

Q13. How often do you talk about your allergies with your friends?

- I often talk to my friends about my allergy
- I sometimes talk to my friends about my allergy
- I never talk to my friends about my allergy because I am embarrased
- I never talk to my friends about my allergy because I don’t know how

Q14. What have been the main effects of your having an allergy? Please tick all that apply (if applicable).

- I carry an adrenaline injector
- My parents have become overprotective of me
- I found it difficult moving away from home
- I have found it difficult making friends
- I have found it difficult socialising and going out with friends
- I have found it difficult getting a boyfriend/ girlfriend
- Difficulty travelling/ going on overnight trips
- Other (please specify):____________

Q15. Thinking about your allergies, what was the hardest thing about moving away from home?

|  |
| --- |

Q16. Do you feel that you need more information about anaphylaxis, or not?

- Yes
- No
- Don’t know

  Q17. Which of the following, if any, would you find useful?
- Please rank each of the following where 1 = most useful and 6 least useful.

  ____ Medical information surrounding anaphylaxis

____ Downloadable resources such as mobile phone applications

____ Someone to talk to (ie meet up face to face) about your concerns

____ Web-based services for young people living with anaphylaxis

____ An online forum (where young people can talk to others with similar severe allergies)

____ General tips and advice to deal with severe allergies.

Q18. Where would you go for information about anaphylaxis?

Please select all that apply.

- The Anaphylaxis Campaign
- NHS website
- Friends/ family
- School nurse
- Allergy Clinic
- Your local GP
- Other (please specify):____________
- None of these

Q19. What are the key things about anaphylaxis that you feel you need more information on?

- My adrenaline injector pen
- Food labelling
- Eating out
- Travelling
- Managing my allergy independently without the help of my parents
- Other (please specify)____________
- None of these

If you have any other comments relating to your experiences of living with anaphylaxis, or about any of the issues covered in the survey, then please write them in the box below.

|  |
| --- |
